# Supplementary material for: Dynamic transcriptomic profiles of zebrafish gills in response to zinc supplementation
Source: BMC Genomics. 2010 Oct 11;11:553. doi: 10.1186/1471-2164-11-553 (PMC3091702; doi:10.1186/1471-2164-11-553)
Supplement: Additional file 2 — Interactive Direct Interaction Network representing the molecular interactions between zinc, copper, iron, calcium and proteins encoded by transcripts changed by zinc supplementation. Mini web-site containing index.html and hyperlinked pages in subdirectory describing a Direct Interaction Network automatically generated based on curated interactions contained within the proprietary PathwayArchitect database. Ovals represent proteins and the circles symbolize metal ions. Objects are coloured by their abundance in zebrafish at the time-point they were significantly different from the control is a scale from -4 fold (dark green) to +4 fold (dark red). Where significant differences were found at more than one time-point, the colour overlay shows expression at the first instance. Dark blue squares denote 'binding', and light blue squares 'expression'; green squares stand for 'regulation', green diamonds for 'metabolism', and green circles for 'promoter binding'. Arrow heads indicate directionality of the interaction where annotated. All nodes and edges can be further interrogated by selecting the relative area of the image. [file 1471-2164-11-553-S2.zip › PathwayArchitect Zn xs DIN/159647.html]

# PROTEIN: GLI3

|  |  |
| --- | --- |
| Name | GLI3 |
| Type | PROTEIN |
| Description | GLI-Kruppel family member GLI3 (Greig cephalopolysyndactyly syndrome) |
| Note | This gene encodes a protein which belongs to the C2H2-type zinc finger proteins subclass of the Gli family. They are characterized as a transcription factor that binds to DNA through zinc finger motifs. These motifs have conserved H-C links. Gli family zinc finger proteins are mediators of Sonic hedgehog (Shh) signaling. The protein encoded by this gene localizes in the cytoplasm and activates patched Drosophila homolog (PTCH) gene expression. It is also thought to play a role during embryogenesis. The encoded protein is associated with several phenotypes- Greig cephalopolysyndactyly syndrome, Pallister-Hall syndrome, preaxial polydactyly type IV, postaxial polydactyly types A1 and B. |
| Alias | AU023367 |
|  | PHS |
|  | add |
|  | PAP-A |
|  | oncogene GLI3 |
|  | DNA-binding protein |
|  | Bph |
|  | PAPA1 |
|  | zinc finger protein GLI3 |
|  | Xt |
|  | AI854843 |
|  | GLI-Kruppel family member GLI3 (Greig cephalopolysyndactyly syndrome) |
|  | GLI3 |
|  | GCPS |
|  | anterior digit pattern deformity |
|  | polydactyly Nagoya |
|  | PAPB |
|  | PAPA |
|  | GLI-Kruppel family member GLI3 |
|  | ACLS |
|  | Pdn |
|  | PPDIV |
|  | extra toes |
|  | Gli3 |
|  | Zinc finger protein GLI3 |
|  | brachyphalangy |


---

|  |  |
| --- | --- |
| GO Component | nucleus |
|  | transcriptional repressor complex |
|  | cytoplasm |


---

|  |  |
| --- | --- |
| GO ID | GO:0030324 |
|  | GO:0003677 |
|  | GO:0006355 |
|  | GO:0000060 |
|  | GO:0005737 |
|  | GO:0007420 |
|  | GO:0017053 |
|  | GO:0005515 |
|  | GO:0008270 |
|  | GO:0007224 |
|  | GO:0003700 |
|  | GO:0009653 |
|  | GO:0005634 |
|  | GO:0009953 |
|  | GO:0007165 |
|  | GO:0007417 |
|  | GO:0030326 |
|  | GO:0006366 |
|  | GO:0003676 |
|  | GO:0046872 |
|  | GO:0016481 |
|  | GO:0006350 |
|  | GO:0007275 |
|  | GO:0016564 |


---

|  |  |
| --- | --- |
| MIM | MIM:200990 |
|  | MIM:146510 |
|  | MIM:174700 |
|  | MIM:165240 |
|  | MIM:174200 |
|  | MIM:175700 |


---

|  |  |
| --- | --- |
| Connectivity | 147 |


---

|  |  |
| --- | --- |
| Entrez ID | 140588 |
|  | 14634 |
|  | 2737 |


---

|  |  |
| --- | --- |
| Agilent ID | A\_53\_P176290 |
|  | A\_14\_P138622 |
|  | A\_14\_P129333 |
|  | A\_51\_P171286 |
|  | A\_14\_P130974 |
|  | A\_53\_P108289 |
|  | A\_53\_P174483 |
|  | A\_44\_P294808 |
|  | A\_14\_P117092 |
|  | A\_51\_P410070 |
|  | A\_23\_P111531 |
|  | A\_51\_P171288 |
|  | A\_14\_P119143 |
|  | A\_23\_P111532 |
|  | A\_53\_P158914 |


---

|  |  |
| --- | --- |
| Cellular Localization | Cytoplasm |
|  | Nucleus |
|  | Cell |
|  | Organelle |


---

|  |  |
| --- | --- |
| DbXref | KEGG pathway##04340##Hedgehog signaling pathway##http://www.genome.jp/dbget-bin/show\_pathway?mmu04340+14634 |
|  | KEGG pathway##04340##Hedgehog signaling pathway##http://www.genome.jp/dbget-bin/show\_pathway?hsa04340+2737 |


---

|  |  |
| --- | --- |
| Pathway | Hedgehog Signaling |
|  | Zn xs inventory |
|  | Zn xs DIN |


---

|  |  |
| --- | --- |
| GO Process | protein-nucleus import, translocation |
|  | dorsal/ventral pattern formation |
|  | smoothened signaling pathway |
|  | regulation of transcription, DNA-dependent |
|  | transcription |
|  | transcription from RNA polymerase II promoter |
|  | lung development |
|  | signal transduction |
|  | brain development |
|  | central nervous system development |
|  | negative regulation of transcription |
|  | development |
|  | embryonic limb morphogenesis |
|  | morphogenesis |


---

|  |  |
| --- | --- |
| UniGene | Hs.199338 |
|  | Rn.92397 |
|  | Mm.5098 |
|  | Rn.103542 |


---

|  |  |
| --- | --- |
| Affymetrix Probeset ID | 101182\_at |
|  | 106173\_at |
|  | 131796\_at |
|  | 137022\_at |
|  | 1444043\_at |
|  | 1450525\_at |
|  | 1455154\_at |
|  | 1456067\_at |
|  | 1569342\_at |
|  | 205201\_at |
|  | 40358\_at |
|  | 73380\_at |
|  | g13518031\_3p\_at |
|  | Hs2.382605.1.S1\_3p\_at |
|  | M57609\_at |
|  | X95255\_s\_at |
|  | 1380776\_at |
|  | 1382077\_at |
|  | 223177\_at |
|  | 1392235\_at |
|  | 1457143\_at |
|  | rc\_AA944438\_at |
|  | Hs.237225.0.S1\_3p\_at |
|  | RC\_H96287\_at |
|  | TC41001\_at |


---

|  |  |
| --- | --- |
| GO Function | transcriptional repressor activity |
|  | protein binding |
|  | transcription factor activity |
|  | DNA binding |
|  | zinc ion binding |
|  | nucleic acid binding |
|  | metal ion binding |


---

|  |  |
| --- | --- |
| Nucleotide | AC005158 |
|  | AK158261 |
|  | AK045960 |
|  | AB073718 |
|  | M20674 |
|  | XM\_225411 |
|  | AC005026 |
|  | AK144851 |
|  | NM\_000168 |
|  | X95255 |
|  | AK037232 |
|  | M57609 |
|  | AK048009 |
|  | AK053922 |
|  | AF418601 |
|  | NM\_008130 |
|  | AJ250408 |
|  | AC005028 |


---

|  |  |
| --- | --- |
| Protein | NP\_000159 |
|  | BAE26100 |
|  | AAA52564 |
|  | AAP21869 |
|  | XP\_225411 |
|  | AAS02015 |
|  | NP\_032156 |
|  | CAA64543 |
|  | BAB71724 |
|  | BAE34433 |
|  | AAS01998 |
|  | P10071 |
|  | Q61602 |
|  | CAB59315 |


---

|  |  |
| --- | --- |
| Organism | Mammal |


---

|  |  |
| --- | --- |
| Location | chromosome 13, 13 14.0 cM, 13 A2 (Mus musculus) |
|  | 13 14.0 cM (Mus musculus) |
|  | chromosome 17, 17q12.1 (Rattus norvegicus) |
|  | chromosome 7, 7p13 (Homo sapiens) |


---

|  |  |
| --- | --- |
